# Educational Films

**Published:** 1921-06-04

**Authors:** 


### Educational Films.

A writer in the *Teacher's World* discusses a question that was considered in these columns some weeks ago—the place of the cinematograph in the school. We have never suggested that the film may not be of very great educational service. On the contrary, we have urged its general usefulness, under proper conditions, in the schools, as well as for the particular purpose of conveying many valuable health lessons in a direct and impressive manner. What we have pointed out in commenting on the dangers of the unrestricted attendance of children at indiscriminate picture-house entertainments, is the weakness of the argument of those who maintain that in the affections of children the school cinematograph can be made to take the place of the picture-house. That suggestion reaches the limit of improbability, and it could only become a fact if the school film were put to quite improper uses.

All teachers who have experimented with films are certainly agreed on these elementary but fundamental points:—

(1) That films are most useful when they are supplementary to the ordinary classroom teaching.

(2) That the needs of the picture-house and the needs of the school are widely different.

(3) That films specially constructed or specially edited for school use are therefore necessary.

(4) That films should be closely correlated to the school curriculum.

(5) That the value of a film is enhanced tenfold if accompanied by explanatory comments skilfully delivered.

The writer quoted above gives an interesting account of the many points to be kept in view in the making of educational films, and of the difficult practical problems that have to be surmounted before a film can be adapted to the needs of the classroom. For instance, it is necessary to decide first of all exactly the type of pupil for which a film is designed, since this regulates entirely the matter to be included and the manner in which it is to be presented.

The next consideration is the accuracy of the facts. This frequently offers grave perplexity, since experts and statistics differ widely.

In writing the scenario, it is essential to study balance, to achieve completeness of treatment without going into unnecessary detail, to make the best use of the limited "footage" available, and to bear in mind from start to finish the particular purpose of the film.

The wording of titles is a feature which calls for considerable skill. The amount of the printed matter should be as small as possible, and the extreme limit in any one title is approximately thirty-five words. The information given in titles should be clear, concise, easily grasped, complete, and suited to the pupils for whom the picture is intended.

The photographic work demands a wide knowledge of cinematograph technique, and great skill in manipulation of the complicated mechanism comprised in a "movie" camera. Ordinary dramatic film photography is child's play compared with the requirements of a good educational film.

When films made on these highly scientific lines are shown, the uninitiated are often heard to exclaim, "How on earth is it done?" The answer of the harassed producer, we are told, would be something like this: "By blood and tears, and unwearying attention to a thousand and one petty details, by inconceivable persistence, unquenchable enthusiasm, ingenuity, and a deep belief in the coming recognition of the film as an indispensable adjunct to present-day educational methods."

